# Emerging Therapeutic Strategies for Targeting Chronic Myeloid Leukemia Stem Cells

**DOI:** 10.1155/2013/724360

**Published:** 2013-07-09

**Authors:** Ahmad Hamad, Zeyad Sahli, Maya El Sabban, Maha Mouteirik, Rihab Nasr

**Affiliations:** Department of Anatomy, Cell Biology and Physiological Sciences, Faculty of Medicine, American University of Beirut, Beirut 1107 2020, Lebanon

## Abstract

Chronic myeloid leukemia (CML) is a clonal myeloproliferative disorder. Current targeted therapies designed to inhibit the tyrosine kinase activity of the BCR-ABL oncoprotein have made a significant breakthrough in the treatment of CML patients. However, CML remains a chronic disease that a patient must manage for life. Although tyrosine kinase inhibitors (TKI) therapy has completely transformed the prognosis of CML, it has made the therapeutic management more complex. The interruption of TKI treatment results in early disease progression because it does not eliminate quiescent CML stem cells which remain a potential reservoir for disease relapse. This highlights the need to develop new therapeutic strategies for CML to achieve a permanent cure, and to allow TKI interruption. This review summarizes recent research done on alternative targeted therapies with a particular focus on some important signaling pathways (such as Alox5, Hedgehog, Wnt/b-catenin, autophagy, and PML) that have the potential to target CML stem cells and potentially provide cure for CML.

## 1. Chronic Myeloid Leukemia

Chronic myeloid leukemia (CML) is a clonal myeloproliferative disorder. The immediate cause of CML was discovered in 1960 by Nowell and Hungerford who described the presence of a small chromosome in the tumor cells of patients with CML, named Philadelphia (Ph) chromosome after the hometown of its discovery [1]. In 1973, Rowley showed that this abnormal Philadelphia chromosome was a result of a reciprocal translocation between chromosome 9 and chromosome 22 [2]. Later, it was shown that a large part of the abelson (abl) gene on chromosome 9 is translocated to the breakpoint cluster region (bcr) gene on chromosome 22 creating *bcr-abl,* a hybrid oncogene coding for the BCR-ABL fusion protein. BCR-ABL is a constitutively active tyrosine kinase leading to the dysregulation of downstream signaling pathways and the increased proliferation and survival of leukemic cells. The discovery of BCR-ABL was a key milestone in understanding CML and devising novel targeted therapies to treat it (reviewed in [3, 4]).

CML is a relatively rare hematopoietic stem cell disorder with an annual incidence of 1-2 cases per 100,000 individuals [5]. Most CML patients are diagnosed with a chronic phase characterized by an uncontrolled proliferation of myeloid elements that retain their ability to differentiate, resulting in an abnormal number of mature granulocytes. Without effective therapy, chronic phase progresses through an accelerated phase into a rapidly fatal acute leukemia known as the blast crisis, characterized by the appearance of immature cells in the blood and a less favorable response to treatment (reviewed in [6]). The mechanisms of CML evolution to blast crisis are complex and may implicate secondary chromosomal changes that may contribute to the malignant phenotype and these include duplication of the Ph chromosome, trisomy 8, and mutations or deletions of tumor-suppressor genes such as p53 or p16. These secondary molecular and chromosomal changes promote increased proliferation, enhanced survival, genomic instability, and arrest of differentiation, a distinctive feature in blast crisis (reviewed in [6]). The acquisition of self-renewal capacity by Granulocyte-macrophage progenitors through the activation of beta-catenin pathway was also shown to occur during the transition of CML from chronic phase to blast crisis [7].

## 2. History of CML Treatment

**Figure 1:**
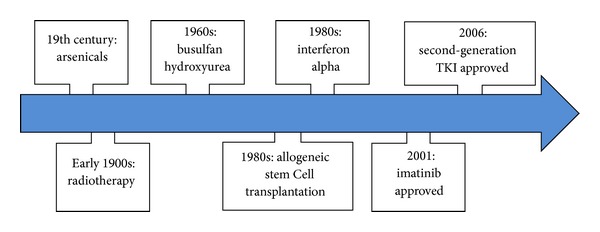
Timeline of CML treatment.

Figure 1 demonstrates the evolution of therapies introduced to treat CML patients throughout the years. The use of arsenic was the only well-documented therapy for CML in the nineteenth century. Despite some toxicity, several preparations of arsenic continued to be used for the treatment of CML until the introduction of radiotherapy in the early 1900s. Then, the introduction of busulfan and hydroxyurea largely replaced radiotherapy in the 1960s. However, these treatments did not have the capacity to improve survival or to induce Ph negativity [8]. Later, in 1980s, allogeneic stem cell transplantation became the only curative treatment for CML but at a significant cost in mortality. Moreover, due to the unavailability of donors, allogeneic stem cell transplantation was only offered to a limited number of patients. Interferon alpha was also introduced in the 1980s to patients ineligible for transplant. Interferon progressively replaced both busulfan and hydroxyurea in the management of CML. It resulted in improved survival and durable cytogenetic responses in approximately one-third of the patients. In 1998, the era of Tyrosine Kinase Inhibitors (TKI) began thereby replacing the two main treatment options that existed for CML previously [9]. The development of these targeted therapies overcame limitations faced by prior conventional treatments. The discovery of TKI had an impact not only on the survival of patients with CML but also on the treatment of other cancers, on the health systems as well as on the scientific research in general [10].

## 3. Evaluation of the Therapeutic Response to TKI

**Figure 2:**
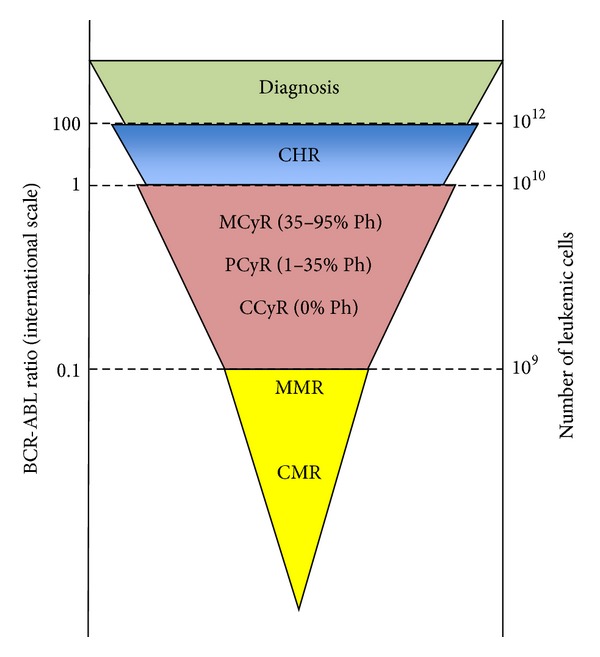
Monitoring response to therapy in CML. Three levels of disease control can be defined in CML: Complete Hematologic Response (CHR), Cytogenetic Response (CyR) (Minor, Partial or Complete) and Molecular Response (MR) (Major or Complete) (Adapted from [33]).

For many diseases, the ultimate measure of the effectiveness of therapy is survival. However, for a disease with a long-term survival such as CML, monitoring tools and markers are needed to allow an early assessment of efficacy or failure. Three levels of disease control can be defined in CML (Figure 2):

1. complete hematologic response (CHR), which is defined as normalization of blood counts and absence of splenomegaly;
2. cytogenetic response (CyR), which is divided into groups according to the percentage of Ph-positive bone-marrow of 20 bone marrow metaphases:
  - complete cytogenetic response (CCyR): 0% Ph chromosome
  - partial cytogenetic response (PCyR): between 1 and 35% of Ph chromosome,
  - minor cytogenetic response (MCyR): between 35 and 95% of Ph chromosome,
3. molecular response (MR), which is defined as bcr-abl transcript level quantitated by real-time PCR using the International scale standardized baseline:
  - major molecular response (MMR): transcript level of 0.1% or less (≥3 log reduction in bcr-abl transcripts),
  - complete molecular response (CMR): No bcr-abl transcript is detectable by real-time PCR.

According to the European LeukemiaNet (ELN) in 2009 [11].

- Optimal response to imatinib requires a CHR within three months; a PCyR within six months and CCyR at 12 months and a MMR at 18 months.
- Failure of imatinib treatment results in no CHR at 3 months, less than PCyR at 12 months and no MMR at 18 months.

## 4. BCR-ABL Inhibitors

### 4.1. First-Generation TKI

Imatinib, a first-generation TKI (formerly STI571), transformed CML from a deadly disease to a chronic, but manageable, one. Imatinib is a 2-phenylaminopyrimidine compound that inhibits the BCR-ABL oncoprotein found in CML cells [12]. It acts by competitively inhibiting the adenosine triphosphate (ATP) binding to the catalytic site of the ABL kinase. The BCR-ABL oncoprotein is considered an ideal target for imatinib in CML patients, since it is present in almost all leukemic cells and absent in normal cells. Thus, imatinib is effective in patients who show Ph chromosome positivity. Due to its high efficacy evaluated in Phase I and II clinical trials, imatinib received an accelerated FDA approval in May 2001 for Ph+ CML patients in blast crisis, accelerated phase, or in chronic phase after failure of treatment with Interferon [13]. In newly diagnosed patients with CML-chronic phase, the International Randomized Study of Interferon and STI571 (IRIS) demonstrated the good tolerability and the superiority of imatinib compared to Interferon in terms of hematologic and cytogenetic responses and frequency of progression to accelerated or blast phases of CML [14]. The key to this high efficiency lies in the specificity of the drug. Imatinib is minimally harmful to normal cells and therefore a clear advantage of quality of life was obtained with imatinib. A long-term follow-up of these patients showed that responses to imatinib are durable. A daily dose of 400 mg imatinib, administered orally, is recommended in first-line therapy for patients with CP CML [15]. However, treatment with imatinib presents some drawbacks, and, based on the results of 8-year follow-up of the IRIS study, only 55% initially enrolled in the imatinib arm of the study remained on the drug. It is estimated that the failure of this therapy, due to therapy discontinuation for lack of efficacy, toxicity, or other reasons, occurred in a notable proportion of patients. For these patients, escalation of the daily dose of imatinib to 600–800 mg was one of the options in patients with suboptimal cytogenetic response or with resistance [16]. Switching to second-generation TKI was another strategy for overcoming failure of imatinib treatment [17]. Finally, the combination of imatinib with other agents such as Interferon was superior to imatinib alone and resulted in a significant improvement of the results. Indeed, the Spirit Study demonstrated that the combination of pegylated interferon and imatinib yielded the best molecular response rate [18]. 

### 4.2. Second-Generation TKI

The emergence of imatinib resistance, intolerance to treatment, and lack of therapeutic response that happen over time in a notable proportion of patients have all motivated the development of second-generation TKI (Dasatinib, Nilotinib, Bosutinib) (Table 1). 

Numerous clinical studies (ENESTnd, DASISION, BELA) have recently demonstrated efficiency and superiority of second-generation TKI versus imatinib in first-line treatment of CML patients (Table 2). They allow more rapid and deeper responses associated with improved outcomes and significantly decreased the rate of progression to accelerated or blastic phases. Consequently, in addition to imatinib, second-generation TKIs are currently considered options for first-line treatment of newly diagnosed patients with CML [19–21].

Dasatinib (formerly BMS-354825) is a second-generation TKI. Dasatinib binds to the ATP binding site of BCR-ABL with more potency than imatinib. Unlike imatinib, which only binds to the inactive conformation of the ABL kinase domain, dasatinib has the ability to bind to both the inactive and active states of BCR-ABL. Dasatinib has a broad spectrum of action not only on BCR-ABL kinase activity, but also on other oncogenic kinases such as Src family, c-Kit, platelet-derived growth factor receptor (PDGFR), and ephrin-A receptor. Dasatinib acts on most imatinib-resistant Abl mutations but not on T315I. The START-C trial, which assessed the use of dasatinib in imatinib-resistant patients in chronic phase showed that dasatinib 70 mg twice daily was superior to 400 mg twice daily of imatinib [22]. Dasatinib was FDA approved in 2007 as a second-line treatment option for chronic phase CML patients. DASISION (Dasatinib versus Imatinib Study in treatment-Naïve CML patients) is a phase 3 trial comparing treatment with 100 mg of dasatinib versus 400 mg of imatinib in newly diagnosed CML chronic phase patients [22]. This study demonstrated the superiority of dasatinib compared to imatinib as a first-line therapy for newly diagnosed CML-CP patients. An important adverse effect that was associated with dasatinib treatment was pleural effusions that occurred in 14.3% of patients but were successfully managed [23].

Nilotinib (formerly AMN107) is another second-generation TKI that binds only to the inactive conformation of BCR-ABL enzyme. It is more potent in binding the ATP-binding site on the BCR-ABL oncoprotein and has a 20 to 50 times better inhibitory activity compared to imatinib [24]. The recommended dosage is 400 mg twice daily. Nilotinib was FDA approved in 2007 as a second-line treatment option of chronic phase CML patients [25]. In a study conducted by Rosti et al., newly diagnosed chronic phase CML patients treated with nilotinib showed a CCyR in 96% of the cases after 12 months of initial treatment. The molecular response reached 85% of patients after 12 months [25]. The results of the randomized ENESTnd clinical trial also showed that nilotinib was superior to imatinib as a frontline treatment [20]. Nilotinib holds another advantage over imatinib, in being active against several imatinib-resistant mutations with exceptions such as the T315I and Y253H mutations [26]. However, there are complications and side effects associated with nilotinib. Nilotinib has a complicated posology. It should be administered after a 2-hour waiting period on an empty stomach and the patient should wait for an hour after taking the drug before eating. Taking into account that this is done twice a day (two pills of 400 mg twice a day), many patients have trouble adhering to this treatment. Furthermore, an important side effect associated with nilotinib is hyperglycemia hence preventing diabetic patients from being treated with this drug [25]. 

Bosutinib (formerly SKI-606) is a new second-generation oral, dual Src/Abl TKI that has been shown to be more efficient than imatinib against CML cell lines [27]. Promising clinical results were obtained with bosutinib in first-, second-, and third-line CML treatment. The phase 3 clinical trial “Bosutinib Efficacy and Safety in Newly Diagnosed CML (BELA),” compared the response in patients treated with bosutinib as upfront therapy to patients treated with imatinib [21]. Even though, comparing the rate of CCyR at 12 months, bosutinib was comparable to imatinib, the median time to reach the first CCyR appeared significantly earlier in patients treated with bosutinib. The superiority of bosutinib over imatinib was also demonstrated when comparing the rate of MMR at 12 months (41% versus 27%), the median time to achieve MMR, and the frequency of transformation to accelerated and blast phases while on treatment [21]. Bosutinib is active against most of imatinib-resistant mutations except for V299L and T315I (Table 1) [28]. With regard to tolerability and toxicity, bosutinib yielded promising results. Diarrhea and elevated liver enzymes were the predominant side effects of bosutinib [21]. 

### 4.3. Third-Generation TKI

Ponatinib (AP24534) is an orally administered TKI designed to inhibit BCR-ABL with mutations, especially T315I, which confers resistance to other TKI such as imatinib, dasatinib, nilotinib, and bosutinib (reviewed in [29, 30]). Ponatinib inhibits both native and mutated BCR-ABL including M244V, G250E, Q252H, Y253F/H, E255 K/V, F317L, M351T, and F359V [31, 32]. Ponatinib and imatinib mechanisms of binding to BCR-ABL are comparable except for the presence of Ponatinib's characteristic carbon-carbon triple bond, between the methylphenyl and purine groups, which allows it to bind to the T315I mutation without steric interference [32, 33]. The PACE (Ponatinib Ph+ ALL and CML Evaluation) trial has been set up to evaluate the effect of Ponatinib on CML patients that were either resistant or intolerant to dasatinib or nilotinib or with T315I mutation. The trial is currently under study; however, there have been some interim results. These results indicated that the overall rate of MCyR was 49% including 62% of patients harboring the T315I mutation [34]. Chronic phase CML-resistant patients treated with Ponatinib showed CHR in 98% of the cases, MCyR in 72% of patients, and MMR in 44%. Among the group of patients with T315I mutation, 100% had a CHR and 92% had a MCyR [35]. These results showed the advantage that Ponatinib holds against other TKI, which were unable to tackle the T315I mutation. 

## 5. Imatinib Resistance

**Figure 3:**
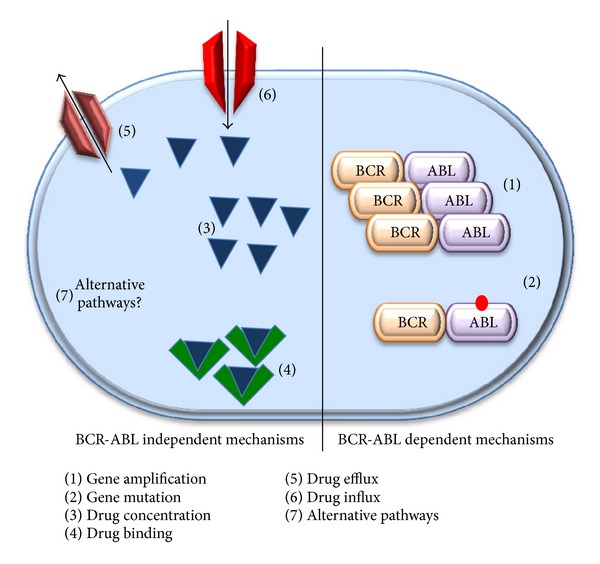
Mechanisms of CML cells resistance to TKI. BCR-ABL dependent mechanisms include (1) duplication or overamplification of the BCR-ABL oncogene that might lead to an elevated ABL kinase activity or (2) BCR-ABL mutations that affect TKI binding. BCR-ABL independent mechanisms deal with complications such as drug concentration (3), sequestration of imatinib in the plasma by the serum protein acid glycoprotein (AGP) or drug binding (4), increased expression of the P-glycoprotein (Pgp) efflux pump or drug efflux (5), and reduced expression of the organic cation transporter hOCT1 or drug influx (6). Other mechanisms that play a role in TKI resistance and CML progression include activation of alternative signaling pathways downstream of BCR-ABL (7).

As mentioned earlier, although imatinib proved to be an excellent treatment option for patients with CML, it was found that the emergence of resistance or intolerance to treatment may affect up to one-third of patients [17]. Some patients may not respond at the beginning of treatment and may never reach a complete hematologic, cytogenetic, or molecular response. This is known as primary resistance to imatinib. Other patients, who initially respond to treatment, may lose response after a certain period of time and this is called secondary resistance [17]. Understanding the underlying causes of resistance is an extremely important step towards combatting the disease. Two main groups of resistance mechanisms exist: BCR-ABL independent mechanisms and BCR-ABL dependent mechanisms (Figure 3). BCR-ABL dependent mechanisms of resistance involve duplication or overamplification of the bcr-abl oncogene that might lead to an elevated ABL kinase activity [36, 37].

Another important mechanism of resistance deals with BCR-ABL mutations. Imatinib can only interact with ATP binding site on the ABL enzyme when it is in its inactive, closed confirmation. Mutations of the binding domain of BCR-ABL occur and affect imatinib-binding leading to resistance [38]. Over 55 types of mutations in the BCR-ABL oncoprotein rendering the binding to imatinib ineffective have been identified. These mutations affect the binding site of imatinib or sites that alter the oncoprotein into its active form to which imatinib cannot bind. The most famous mutation is T315I associated with a substitution of threonine with isoleucine at position 315. This mutation makes it impossible for imatinib to bind the ATP-binding site due to the elimination of an oxygen molecule needed for binding due to steric hindrance. T315I mutation is also known as the gatekeeper mutation (reviewed in [39]).

BCR-ABL independent mechanisms are the second major category of resistance to imatinib. These may lead to a decrease in the intracellular level of imatinib due to complications with drug efflux, drug influx, drug binding, or drug concentration. Examples are increased expression of the P-glycoprotein (Pgp) efflux pump, reduced expression of the organic cation transporter hOCT1, and sequestration of imatinib in the plasma by the serum protein acid glycoprotein (AGP) [40, 41]. BCR-ABL independent activation of signaling such as Src/Ras/Raf/MEK/Lyn, STAT, Wnt/beta catenin, Hedgehog, FoxO, and SIRT1 may also play a role in resistance and CML progression [33]. Interestingly, CML stem cells are another player that can mediate imatinib resistance. CML stem cells are insensitive to imatinib despite BCR-ABL inhibition. This may suggest that BCR-ABL independent mechanisms might contribute to CML stem cells resistance to TKI [42].

## 6. CML Stem Cells

**Figure 4:**
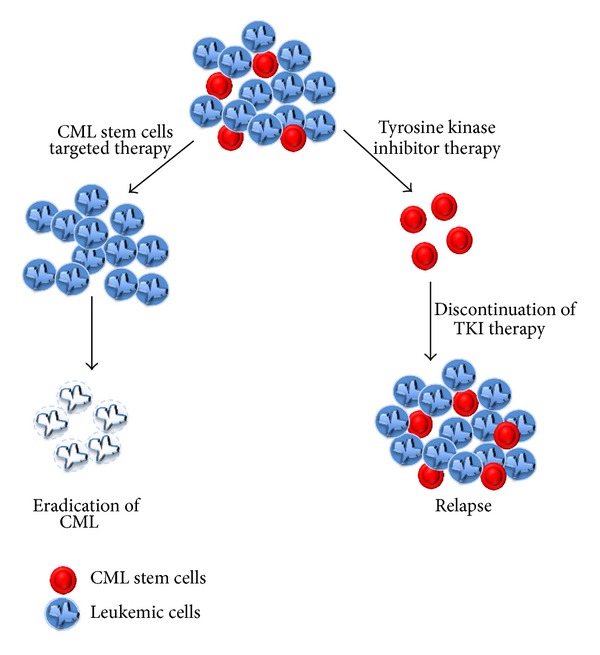
Schematic representation of CML stem cells response to therapy.

CML is a hematopoietic stem cell disorder. The failure of targeted therapy by TKI to cure CML patients despite their ability to induce rapid remission was the first evidence that hinted to the presence of leukemic stem cells in CML [33]. Some data confirmed later the presence of a small population of primitive quiescent leukemic stem cells insensitive to imatinib that sustain the disease and provide a reservoir of leukemic cells (Figure 4) [43–45].

Second-generation TKI has been shown to target progenitors better than imatinib due to their higher affinity to BCR-ABL but these drugs, like imatinib, do not cure the disease and patients still develop resistance to therapy and relapse upon discontinuation of the drug [33, 46]. Moreover, most CML patients in remission continue to show minimal residual disease detected by the quantitative real-time PCR analysis of peripheral blood or bone marrow [42]. The immunophenotypic recognition of a CML stem cell remains elusive. CML stem cells were described as a small subset of cells carrying the phenotype Lin−, CD34+, CD38−, and CD90+ [47]. However, it was also proposed that CML stem cells form only a tiny population of the Lin−, CD34+, CD38−, and CD90+ [48]. CML stem cells are similar to normal hematopoietic stem cells in their ability to self-renew and to give rise to a heterogeneous population of cells but differ by the bcr-abl genetic marker that is specific to CML. CML stem cells exist in a quiescent state and are endowed by a long-term engraftment potential. Recent evidence suggests that CML stem cells are not fully addicted to BCR-ABL and that they are not dependent on this oncoprotein for their survival (reviewed in [43]). This might explain the insensitivity of CML stem cells to TKI and why these cells persist in patients even after several years of TKI treatment (reviewed in [49]). The presence of CML stem cells adds an extra challenge in treating CML patients because it creates a new target to hit and eliminate. Alternative targeted therapies will be needed, which either alone or in combination with TKI will lead to the suppression of CML stem cells. This may suggest that signaling pathways essential for CML stem cells survival should be identified as potential targets for therapy. 

## 7. Molecular Pathways in CML Stem Cells

**Figure 5:**
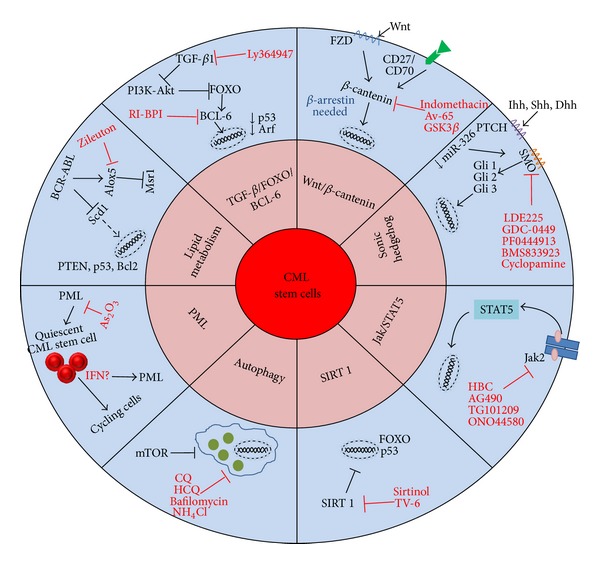
Alternative signaling pathways for overcoming resistance of CML Stem Cells against tyrosine kinase inhibitors. Sonic hedgehog (Shh), Indian hedgehog (Ihh), Desert hedgehog (Dhh), Smoothened (Smo), STAT5 (signal transducer and activator of transcription), retroinverso BCL6 peptide inhibitor (RI-BPI), chloroquine (CQ), hydroxychloroquine (HCQ), Sirtuin 1 (SIRT1), tenovin-6 (TV-6), Arachidonate 5-lipoxygenase (*Alox5*), stearoyl-CoA desaturase 1 (Scd1), promyelocytic leukemia protein (PML), arsenic trioxide (As_2_O_3_), and Interferon alpha (IFN).

Many pathways have been studied in order to understand how CML stem cells survive and function and to find the signaling pathway that if inhibited will lead to the eradication of CML stem cells or their sensitization to TKI or other antileukemic drugs. Of these candidate pathways, the most attractive have been the *Alox5* pathway, the sonic hedgehog pathway (SHH), the Wnt/*β*-catenin pathway, the JAK/STAT pathway, the TGF-Beta/FOXO/BCL-6 pathway, among others (Figure 5).

### 7.1. *Alox5*/Lipid Metabolism

A new role for lipid metabolism in CML stem cells maintenance has recently emerged. Arachidonate 5-lipoxygenase (*Alox5*) is part of the 5-LO pathway that synthesizes Leukotriene B4 (LTB4) [50]. *Alox5* is primarily upregulated in CML stem cells. Mice with *Alox5* knockout in LSC failed to develop CML suggesting the critical role of *ALOX5* in CML leukemogenesis. Importantly, *Alox5* deficient HSC have normal functioning suggesting that *Alox5* may not be critical for their development [51]. In murine CML cells, *Alox5* gene was not affected by imatinib treatment indicating that its upregulation does not require kinase activity. Treatment of CML mice with Zileuton, an *Alox5* enzymatic activity inhibitor, depleted murine CML stem cells and prolonged the survival of CML mice [51]. Combined administration of Zileuton and Imatinib was seen to be more effective on CML mice survival than either drug alone. Pharmacological inhibition of *Alox5* produced promising data in murine CML therapy and Zileuton is currently in a Phase I study in combination with imatinib in CP CML patients. Dissection of this pathway revealed that *Alox5* functions through Msr1 downregulation by BCR-ABL. Msr1 is an important regulator of the PI3k-AKT pathway and *β*-catenin and accordingly affects CML stem cells function and CML development [51]. The same group also demonstrated that stearoyl-CoA desaturase 1 (Scd1), another regulator of lipid metabolism, is downregulated in CML stem cells. Scd1 deletion accelerated leukemia development in the CML mouse model through targeting of leukemic stem cells function but not that of normal HSC. On the contrary, Scd1 overexpression resulted in a delay of CML development indicating its role as tumor suppressor in CML leukemogenesis [52]. Effective therapeutic strategies to inhibit Alox-5 or induce scd1 expression can be promising approaches to specifically eradicate CML stem cells.

### 7.2. TGF-Beta/FOXO/BCL-6

The PI3 K/AKT pathway is one of the signaling pathways activated by BCR-ABL that leads to the phosphorylation, cytoplasmic retention, and inactivation of the forkhead transcription factor FOXO. Inhibition of FOXO is important for the increased proliferation and decreased apoptosis of CML cells. FOXO transcription factors are also critical for CML stem cells maintenance. Recent work, using the CML mouse model, demonstrated that drug resistance in CML stem cells is due to TGF-*β* secreted by their microenvironment. TGF-*β* inhibits AKT activation and leads to the release of the inhibitory sequestration of FOXO and its activation promoting the quiescence of CML stem cells. Accordingly, inhibiting TGF-*β* signaling pathway might lead to the reduction of CML stem cells that are currently resistant to TKI [53]. Treatment of human CML stem cells with a TGF-*β* inhibitor (LY364947) inhibited their clonogenic activity *in vitro* [53]. Furthermore, FOXO3a deficiency decreased the ability of murine CML stem cells to cause disease. In fact, FOXO3a deficiency in combination with TGF-*β* inhibition and imatinib led to the depletion of murine CML stem cells [53]. It was also shown that Bortezomib inhibited BCR-ABL-induced proteasome-dependent degradation of FOXO and led to a regression of CML in an *in vivo* mouse model [54].

BCL-6, an important downstream effector of FOXO that mediates the repression of Arf and p53, is critical for the survival and self-renewal of CML stem cells [55]. BCL-6 inhibition induces CML stem cells to exit quiescence, leaving them more sensitive to TKI inhibition.

These results provide evidence that TGF-*β*-FOXO-BCL-6 pathway is a potential therapeutic target in CML. Pharmacological inhibition of TGF-*β* (by Ly364947) or of BCL-6 (by RI-BPI) may represent an efficient strategy to deplete CML stem cells. 

### 7.3. JAK/STAT

BCR-ABL protein activates several signaling pathways, including the JAK/STAT pathway that stimulates cell proliferation, differentiation, and cell migration. The signal transducer and activator of transcription 5 (STAT5) is a downstream effector of BCR-ABL; it is constitutively activated due to its phosphorylation by BCR-ABL [56, 57]. STAT5 was validated as a therapeutic target for CML after the discovery that murine CML did not develop in mice lacking STAT5 [58]. Inhibition of STAT5 phosphorylation has been shown to be an interesting target for eliminating leukemic stem cells [56].

JAK2 is also activated in CML, but its role is not totally understood. Inhibition of JAK2 signalling reduced BCR-ABL and other downstream oncogenic signaling pathways [59]. Several inhibitors of JAK2 have been developed since its inhibition overcomes imatinib resistance by inducing apoptosis in imatinib-resistant cell lines (including those harboring T315I cells). AG490, a potent and specific JAK2 inhibitor reduced BCR-ABL-induced oncogenicity and inhibited cell survival of imatinib-sensitive CML cell lines. AG490 induced apoptosis also in imatinib-resistant CML cell lines expressing the famous T315I mutation [60]. Other JAK2 inhibitors such as TG101209 and HBC were shown to have clinical efficacy against CML cell lines, and, in combination with imatinib, HBC significantly induced apoptosis in CML-BC cells. A new dual kinase inhibitor for JAK2 and ABL kinases called ON044580 was recently discovered and was shown to target both imatinib-sensitive and resistant K562 CML cells. By contrast, it has been shown in a recent study that JAK2 is dispensable for CML cell survival and maintenance *in vitro* and *in vivo* [57]. Given the controversial findings about the importance of JAK2 in CML, further research is still needed to confirm its validity as a therapeutic target.

### 7.4. Wnt/*β*-catenin

Canonical Wnt/*β*-catenin signaling is another signaling pathway that plays a major role during embryogenesis (reviewed in [61]). *β*-catenin represents the central downstream effector of the canonical Wnt signaling pathway. The canonical pathway can be activated in several ways. Wnt ligands bind to Frizzled and LRP6 receptors. This results in *β*-catenin stabilization and nuclear translocation [62]. *β*-catenin is also central to cadherin CD27-CD70 signaling [63]. Cadherins mediate cell adhesion through homotypic interaction between cell surface receptors leading to *β*-catenin stabilization and linkage to actin cytoskeleton. *β*-catenin pathway influences normal stem cell abilities to self-renew [64]. The Wnt pathway plays an important role in CML stem cells. In CML, this pathway is aberrantly activated. It fuels leukemic stem cells and drives them towards excessive self-renewal, and it has also been implicated in blast crisis evolution [65, 66]. Genetic inactivation of the *β*-catenin gene impairs the self-renewal of BCR-ABL-induced CML without affecting disease development in primary recipients [50]. The use of indomethacin, which enhances the degradation of active *β*-catenin, led to reduction in CML stem cells numbers. Another novel Wnt/*β*-catenin inhibitor, AV65, was shown to inhibit proliferation and induce apoptosis of CML cell lines even those harboring the T315I mutation. The cause of *β*-catenin overexpression in CML stem cells is unclear but may be attributed to its stabilization due to its reduced degradation related to GSK3*β* inactivation downstream of BCR-ABL. *β*-catenin overexpression is also observed with CD27-CD70 interaction [63]. CD27 is a TNF receptor that is expressed on murine CML stem cells and progenitors. The binding of CD27 to its ligand CD70 induces the overexpression of Wnt target genes leading to increased proliferation and differentiation of CML stem cells. Blocking CD70-CD27 interactions in CML mice resulted in delayed CML progression and prolonged survival of CML mice [63].

In the Wnt pathway, *β*-arrestin2, a scaffold protein that functions in G protein-coupled receptor (GPCR) signaling regulation, has been shown to be required for the activation of *β*-catenin in mouse embryonic fibroblasts. Loss of *β*-arr2 led to a significant reduction in activated *β*-catenin levels leading to a decrease in the number of normal stem cell colonies and reduction in their ability to self-renew. *β*-arr2 signaling is essential for CML initiation and progression *in vitro* and *in vivo*. *β*-arr2 inhibition prevented the establishment as well as development of the blast crisis phase of CML in mice [67]. Importantly, deletion of *β*-arr2 did not affect normal hematopoiesis representing a valid therapeutic target in CML.

### 7.5. Autophagy

Autophagy is a genetically controlled cellular recycling process. It functions in lysosomal mediated organelle recycling such as mitochondria removal, preventing damage from reactive oxygen species, protein degradation, and adaptation by providing an alternative source of energy in starvation conditions. BCR-ABL signaling activates mTOR, an inhibitor of autophagy. TKI treatment induces both apoptosis and autophagy [68]. Imatinib was shown to reduce the expression of microRNA-30a, a potent inhibitor of autophagy by targeting Beclin 1 and ATG expression [69]. The inhibition of BCR-ABL in CML stem cells (CD34+CD38−) may lead to the activation of the autophagy pathway. Normal stem cells would be spared, as pharmacological inhibition of autophagy alone has modest or no effects on normal or CML progenitors [70]. The initiation of autophagy serves as a protection mechanism for CML stem cells against TKI-mediated apoptosis. The combination of imatinib and Chloroquine (CQ), an inhibitor of autophagy, eliminates CML stem cells in long-term culture assays [70, 71]. Inhibition of autophagy can also restore CML stem cells sensitivity to TKI. Bafilomycin A1, a vacuolar-type H-ATPase inhibitor, and chloroquine (CQ), hydroxychloroquine (HCQ), and NH_4_Cl, which all inhibit the formation of autophagosomes, sensitized CML cell lines, including those carrying resistant BCR-ABL mutants, to imatinib [68, 70]. Knockdown of the autophagy genes *Atg*5 and *Atg*7 in K562 and primary CML cells enhanced TKI-induced cell death [68, 70]. Currently CHOICES (CHlOroquine and Imatinib Combination to Eliminate Stem cells), the first clinical trial to use autophagy inhibition in CML treatment, is in its phase II [68].

### 7.6. Sonic Hedgehog

The hedgehog pathway is a prominent signaling pathway active during embryogenesis. In adult life, the activity of this pathway is retained physiologically in stem cells and pathologically in cancer cells (reviewed in [72, 73]). The hedgehog proteins exist in three isoforms: the sonic hedgehog (Shh), the Indian hedgehog (Ihh), and the desert hedgehog (Dhh) (reviewed in [72, 73]). After their secretion, hedgehog proteins bind to Patched (Ptch) leading to the release of Smoothened (Smo) from Ptch binding and resulting in activation of Gli transcription factors (Gli1, Gli2, and Gli3) which in turn modulate cell proliferation and survival (reviewed in [74]). The hedgehog pathway is intimately related to normal and malignant hematopoiesis due to its role in controlling the proliferation/differentiation balance in normal and leukemic stem cells [72, 75]. Several studies have linked aberrant activation of the hedgehog pathway to CML. Furthermore, the hedgehog pathway has been shown to activate and regulate BCR-ABL in a hierarchal fashion [74, 76]. Another study has shown that hedgehog pathway proteins (such as SHH, SMO, and GLI1) and their downstream effectors are upregulated in CML patients in comparison to normal subjects, and the same proteins are higher in blast crisis patient's cells than in chronic phase cells suggesting a key role that the hedgehog pathway might play in CML blastic transformation of CML patients [77]. The importance of the Shh pathway and, in particular, SMO and PTCH1 expression was highlighted in a study correlating levels of expression with CML disease progression. Targeting the hedgehog proteins or any of their downstream effectors might be a promising way to eliminate CML stem cells as long as the developed inhibitors do not affect normal hematopoietic cells. Currently several drugs are being tested including Smo inhibitors such as cyclopamine that was shown to selectively target CML stem cells while sparing normal hematopoietic stem cells [72]. Upregulation of Smo in CD34+ CML cells was correlated with downregulation of microRNA-326. Restoration of microRNA-326 level that targets the signal transducer Smo could be an alternative future strategy to eradicate CML CD34+ stem cells through the Hedgehog pathway [78]. The clinical efficacy of other Smo inhibitors such as GDC-0449, LDE225, and BMS833923 or PF0444913 and GLI proteins inhibitors such as GANT 61 is being currently explored. 

### 7.7. SIRT1

BCR-ABL acts through STAT5 to upregulate Sirtuin 1 (SIRT1), a NAD+-dependent protein deacetylase [79, 80]. SIRT1 promotes mammalian cell survival, DNA repair, cell cycle, and metabolism under environmental stresses [79, 80]. Normal adult hematopoietic stem cells and progenitors express low levels of SIRT1. Upregulation of SIRT1 is detected in CD34+chronic CML progenitor cells and increases in later stages of CML [79]. SIRT1 plays an important role in myeloid leukemogenesis and in CML stem cells resistance to imatinib. p53 is an important factor in mediating SIRT1 effects. Imatinib treatment of CML cell lines partially decreased SIRT1 levels [80]. The decrease in SIRT1 expression was not observed in imatinib-resistant T315I mutant BCR-ABL. A SIRT1 inhibitor, Sirtinol, increased apoptosis in CML cell lines. Importantly, *SIRT1*-deficient cells showed a significantly delayed disease development in mice while knockdown of* SIRT1* in normal progenitor did not affect mice survival [79]. Controversial findings were reported with the combination of imatinib and SIRT1 inhibition. Although tenovin-6 (TV-6), a small molecule SIRT1 inhibitor, sensitizes mice and human CML progenitor cells to imatinib-induced apoptosis, there was no increase in survival with TV-6 combined with imatinib compared with single drug treatment. In another study, combined treatment of imatinib and TV-6 increased apoptosis in CML stem and progenitor cells including those with T315I mutation compared to either agent alone. Therefore, the role of SIRT1 inhibition as targeted therapy to overcome CML drug resistance warrants more investigation.

### 7.8. PML

 The promyelocytic leukemia protein (PML), an essential component of PML nuclear bodies, has been shown to have a critical role in apoptosis, proliferation, senescence, and HSC maintenance. PML is deregulated in CML and is highly expressed in bone marrow from CP CML patients [81]. An inverse correlation was described between PML expression and the rate of CCyR and CMR of these patients. PML is also critical for CML stem cells maintenance. PML makes CML stem cells dormant and resistant to therapy. In fact, BCR-ABL^+^ PML-deficient cells failed to induce leukemia in mice. Since high levels of PML correlate with poor prognosis, arsenic trioxide, which induces the degradation of PML protein, is an ideal compound for CML therapy. Arsenic downregulates PML expression and forces murine CML stem cells to enter the cell cycle consequently making them more sensitive to therapy. Accordingly, the combination of arsenic and Ara-c in murine CML induced apoptosis in the leukemic stem cells compartment. A phase I clinical trial (NCT01397734) is ongoing to evaluate disease response after combined therapy arsenic trioxide and imatinib, dasatinib, or nilotinib and to assess PML expression in the CML stem cell compartment. We also demonstrated, in a murine transplantation model of CML, that the combination of arsenic and interferon alpha sharply diminished transplantation of CML cells in secondary recipients, pointing to exhaustion of murine CML stem cells (unpublished data). The effect of interferon on CML stem cells is not yet understood. However, it was shown that interferon induces the turnover, proliferation, and possibly the exhaustion of normal hematopoietic stem cells [82]. These studies plea for a clinical exploration of this combination, knowing that interferon and arsenic have both shown clinical activity in CML, alone or in combination with imatinib. 

## 8. Conclusion

Despite the clinical efficacy and the good tolerability of the currently available TKI, many major problems persist: the long-term tolerability, the need for treatment interruption for fertility and pregnancy due to the potential risk to the fetus, the insensitivity of the highly resistant mutation T315I to these TKI and their inability to eradicate CML stem cells and minimal residual disease (MRD). Quiescent CML stem cells escape currently available first- and second-generations TKI. Why this pool of CML stem cells is still resistant to all currently available TKI is still an unresolved issue. This translates into the inability of TKI to cure CML and reflects the need of long-term therapy. Currently, to maintain remission, it is not recommended to discontinue TKI therapy, and patients should only stop TKI therapy in the context of clinical trial [83]. This is due to the need of close molecular monitoring to promptly restart the therapy as soon as molecular recurrence occurs. Another disadvantage for the long-term TKI therapy comes from the high cost of the TKI and the economic burden that these expensive drugs cause on the health care systems. Targeting CML stem cell pathways using a single agent or a combination therapy is an interesting and attractive strategy to cure CML. The identification of driving pathway in CML stem cells that can be targeted could solve the problem of minimal residual disease and potentially cure CML patients. Promising strategies that specifically target CML stem cells are currently being explored to allow discontinuation of TKI and eradication of MRD. 

## Figures and Tables

**Table 1:** Summary of the tyrosine kinase inhibitors and their effects on T315I.

TKI	Originally termed	TKI generation	Acts on T315I
Imatinib	STI571	First	No
Dasatinib	BMS-354825	Second	No
Nilotinib	AMN107	Second	No
Bosutinib	SKI-606	Second	No
Ponatinib	AP24534	Third	Yes

**Table 2:** Summary of the 12-month results of ENESTnd, DASISION, and BELA clinical trials that have recently demonstrated the superiority of second-generation TKI in terms of complete cytogenetic response (CCyR) and major molecular response (MMR) versus imatinib in first-line treatment of CML patients.

Results	DASISION trial		ENESTnd trial		BELA trial	
	Dasatinib	Imatinib	Nilotinib	Imatinib	Bosutinib	Imatinib
CCyR (% after 12 months)	83	72	78	65	70	68
MMR (% after 12 months)	46	28	44	22	41	27
